# *Notes from the Field:* Long COVID Prevalence Among Adults — United States, 2022

**DOI:** 10.15585/mmwr.mm7306a4

**Published:** 2024-02-15

**Authors:** Nicole D. Ford, Abraham Agedew, Alexandra F. Dalton, Jordan Singleton, Cria G. Perrine, Sharon Saydah

**Affiliations:** ^1^Coronavirus and Other Respiratory Viruses Division, National Center for Immunization and Respiratory Diseases, CDC; ^2^General Dynamics Information Technology, Falls Church, Virginia; ^3^Epidemic Intelligence Service, CDC.

**Summary**

**What is already known about this topic?**

Long COVID encompasses a range of health problems that emerge, persist, or recur following acute COVID-19 illness.

**What is added by this report?**

Age- and sex-standardized prevalence of reporting ever having experienced Long COVID among Behavioral Risk Factor Surveillance System survey respondents in U.S. states and territories ranged from 1.9% (95% CI = 0.9%–4.1%) in the U.S. Virgin Islands to 10.6% (95% CI = 9.5%–11.8%) in West Virginia; prevalence of Long COVID exceeded 8.8% in seven states.

**What are the implications for public health practice?**

Ongoing assessment of jurisdiction-specific prevalence of Long COVID could inform policy, planning, or programming to support U.S. adults experiencing Long COVID.

## Introduction

Post-COVID conditions, also known as Long COVID, encompass a range of health problems* that emerge, persist, or recur following acute COVID-19 illness, including fatigue, respiratory symptoms, and neurologic symptoms. In 2022, 6.9% of U.S. adults reported ever experiencing Long COVID (*1*). State- and territory-specific surveillance estimates can guide public health action to mitigate the impact of Long COVID; however, few published data are available. The Association of State and Territorial Health Officials (*2*) and the Council of State and Territorial Epidemiologists (*3*) have published reports outlining gaps and needs in Long COVID surveillance for state, tribal, local, and territorial public health agencies.

## Investigation and Outcomes

CDC analyzed data from noninstitutionalized U.S. adults aged ≥18 years participating in the 2022 Behavioral Risk Factor Surveillance System (BRFSS), a population-based cross-sectional survey (*4*). Respondents were sampled using random digit dialing of both landline and cellular telephones. Self-reported age, sex, previous COVID-19 diagnosis,^†^ and ever having experienced Long COVID were ascertained via telephone interview. Long COVID was defined as the self-report of any symptoms lasting ≥3 months that were not present before having COVID-19. CDC estimated weighted age- and sex-standardized prevalence with a 95% CI of ever having experienced Long COVID among all adults nationally, irrespective of COVID-19 history, in the 50 states, the District of Columbia, Guam, Puerto Rico, and the U.S. Virgin Islands. Estimates were standardized to the 2020 U.S. Census Bureau population of noninstitutionalized, civilian adults. Sex-specific weights by age group were applied for persons aged 18–44, 45–64, and ≥65 years. Analyses were conducted using SAS-callable SUDAAN (version 9.4; RTI International) and account for complex survey design. Prevalence estimates were divided into quintiles. This activity was reviewed by CDC, deemed not research, and was conducted consistent with applicable federal law and CDC policy.^§^

## Preliminary Conclusions and Analysis

Nationally, 6.4% of noninstitutionalized U.S. adults reported ever having experienced Long COVID (95% CI = 6.2%–6.5%) (Supplementary Table, https://stacks.cdc.gov/view/cdc/147385). The weighted age- and sex-standardized prevalence ranged from 1.9% (95% CI = 0.9%–4.1%) for the U.S. Virgin Islands to 10.6% (95% CI = 9.5%–11.8%) for West Virginia (Figure) and exceeded 8.8% (the highest prevalence quintile cutoff) in seven states. Prevalences tended to be lower in New England and the Pacific and higher in the South, Midwest, and West.^¶^

**FIGURE:**
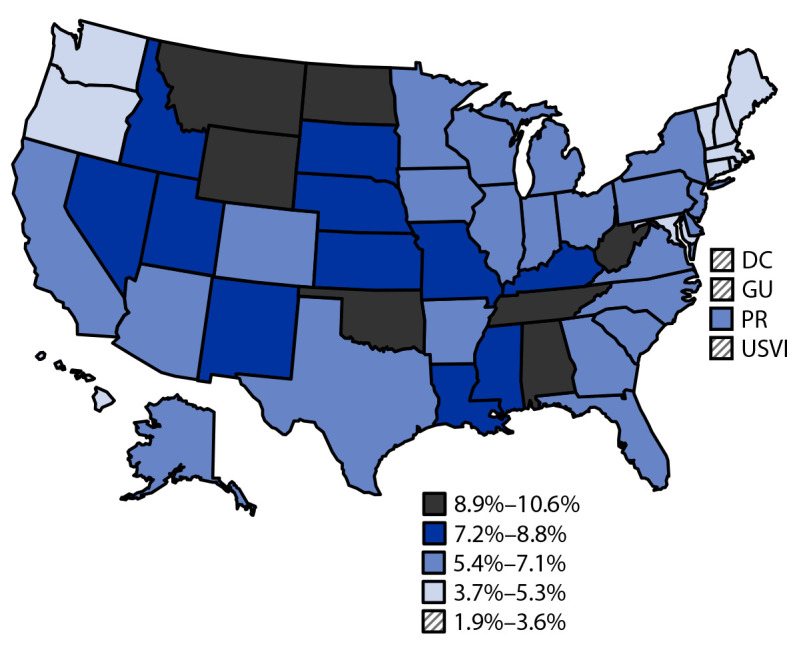
Prevalence of reported experience of Long COVID among adults aged ≥18 years, by jurisdiction — Behavioral Risk Factor Surveillance System, United States, 2022 **Abbreviations:** DC = District of Columbia; GU = Guam; PR = Puerto Rico; USVI = U.S. Virgin Islands.

This study was subject to some limitations. BRFSS did not capture treatment during acute COVID infection, time since COVID-19 illness, or duration or severity of symptoms, which could influence the reported prevalence of Long COVID. In addition, information about COVID-19 vaccination was only available for a subset of jurisdictions and is not included in this report.

The findings in this report address an important data gap in knowledge about the prevalence of Long COVID. Given the increased health care needs among persons experiencing Long COVID (*5*), ongoing assessment of state- and territory-level prevalence data could guide policy, planning, or programming. State-level estimates might also help identify geographic disparities in Long COVID across the United States that could guide interventions to promote health equity.

## Footnotes

*
https://www.cdc.gov/coronavirus/2019-ncov/long-term-effects/index.html

† Respondents were classified as having previously had COVID-19 if they responded affirmatively to the question, “Has a doctor, nurse, or other health professional ever told you that you tested positive for COVID-19?” or if they reported a positive test result based on a home test.

§ 45 C.F.R. part 46.102(l)(2), 21 C.F.R. part 56; 42 U.S.C. Sect. 241(d); 5 U.S.C. Sect. 552a; 44 U.S.C. Sect. 3501 et seq.

¶
https://www.cdc.gov/nchs/hus/sources-definitions/geographic-region.htm
